# Assessing hemodynamic changes in diabetic patients with and without peripheral arterial disease using dynamic vascular optical spectroscopy

**DOI:** 10.1186/s12938-026-01564-z

**Published:** 2026-04-01

**Authors:** Rabah Al abdi, Nesreen Saadeh, Heba Hijazi, Nabil Al-zoubi, Alessandro Marone, Nisha Maheshwari, Andreas H. Hielscher

**Affiliations:** 1 Electrical Computer and Biomedical Engineering Department, College of Engineering, Abu Dhabi University, Abu Dhabi, United Arab Emirates; 2 Biomedical Engineering Department, Faculty of Engineering, Jordan University of Science and Technology, Irbid, Jordan; 3 Department of Internal Medicine, Faculty of Medicine, Jordan University of Science and Technology, Irbid, Jordan; 4 Department of Health Care Management, College of Health Sciences, University of Sharjah, Sharjah, United Arab Emirates; 5 Department of Health Management and Policy, Faculty of Medicine, Jordan University of Science and Technology, Irbid, Jordan; 6 Department of General Surgery and Urology, Faculty of Medicine, Jordan University of Science & Technology, King Abdullah University Hospital, Irbid, Jordan; 7 Department of Biomedical Engineering, Tandon School of Engineering, New York University (NYU), Brooklyn, NY USA

**Keywords:** Peripheral arterial disease (PAD), Diabetes, Non-invasive diagnostics, Blood circulation, Dynamic vascular optical spectroscopy, Hemodynamic biomarkers

## Abstract

**Background:**

Peripheral Arterial Disease (PAD) affects approximately 230 million people globally and is prevalent in 20%-50% of individuals with diabetes. Current diagnostic tools, such as the ankle-brachial index, have limited sensitivity in diabetic populations. This study utilizes dynamic vascular optical spectroscopy (DVOS) at thigh occlusion pressures of 60 and 100 mmHg to examine hemodynamic responses in the feet of diabetic patients, primarily aiming to enhance the detection of PAD in this group. We conducted a prospective cohort study involving 118 diabetic patients with PAD (50 males; age: 59.1 ± 7.9 years) and 118 diabetic subjects without PAD (52 males; age: 54.8 ± 13.7 years). DVOS was used to measure changes in oxyhemoglobin (ΔHbO), hemoglobin flow (HF), and tissue oxygen consumption (VO_2_) in the foot during occlusions. Group differences were assessed using the Mann–Whitney U test, and multivariable logistic regression was used to adjust for age, sex, diabetes duration, and hypertension duration, BMI, and smoking status. Diagnostic performance was measured using ROC analysis with stratified fivefold cross-validation.

**Results:**

The 60-mmHg occlusion revealed clearer hemodynamic differences than the 100-mmHg. During 60-mmHg occlusion, mean values for ΔHbO (2.0 ± 1.74 μmol), HF (0.86 ± 0.49 μmol/s), and VO_2_ (0.040 ± 0.022 mL O_2_/100 mL tissue/min) were significantly lower in the PAD group compared to the non-PAD group (ΔHbO: 6.81 ± 4.11 μmol; HF: 2.48 ± 0.85 μmol/s; VO_2_: 0.066 ± 0.035 mL O_2_/100 mL/min; p < 0.00625). After adjustment for covariates, HF during the 60-mmHg occlusion remained the strongest discriminator for PAD, with an AUC of 0.983, accuracy of 0.918, sensitivity of 0.932, and specificity of 0.904 (mean across folds).

**Conclusions:**

Hemodynamic parameters measured by DVOS during thigh venous occlusion differ significantly between diabetic patients with and without PAD. Our findings suggest that 60-mmHg occlusion is more effective than 100 mmHg for eliciting PAD-sensitive hemodynamic parameters. These results highlight the potential sensitivity of DVOS to PAD-related vascular impairment during low-pressure thigh occlusion.

## Introduction

Diabetes mellitus (DM) affects about one in eleven adults worldwide, with roughly half a billion people living with the disease globally [1]. DM is one of the major risk factors for peripheral artery disease (PAD). In 2021, it was estimated that 230 million people suffered from PAD [2], which is strongly associated with strokes and cardiovascular diseases [3]. The prevalence of DM in Jordanians has almost doubled from 13% in 1994 to 22.5% in 2017 [4]. Additionally, among Jordanian patients with diabetes, the prevalence of PAD is 28.6% [5], highlighting the substantial impact of these diseases in the region.

PAD can be defined as a complete or partial occlusion of one or more of the arteries of the upper or lower limbs, leading to reduced blood flow. In advanced cases, PAD can lead to tissue loss. In diabetes, PAD predominantly affects the distal arteries of the lower limbs. More than 70% of people with PAD are asymptomatic, which delays the diagnosis and treatment of the disease [6]. PAD may progress, reducing blood flow to the lower extremities and potentially leading to complications such as foot ulcerations, infection, tissue loss (gangrene), and, in some cases, lower limb amputation. The risk of developing PAD is particularly high in patients with diabetes and is associated with a worse prognosis compared to non-diabetic individuals [7]. Therefore, regardless of age, the American Diabetes Association advises every diabetic patient to have PAD checked every five years [8]. Early treatment methods for PAD can be relatively easy and low cost, such as lifestyle changes, quitting smoking, control of hyperlipidemia and diabetes, and the use of clopidogrel or aspirin [9]. These interventions can improve the quality of life and disease prognosis.

Currently, the most common method to monitor PAD within the lower extremities is the ankle-brachial index (ABI) [10]. ABI reflects pressure reduction rather than blood flow, it serves as a practical screening tool for identifying hemodynamically significant stenosis. ABI is limited in its capability to diagnose and monitor PAD in patients with incompressible arteries, such as diabetic patients and those with renal insufficiency [11]. Diabetic patients are the largest subpopulation with PAD [7], so there is a substantial unmet clinical need for a better screening tool for them. Other tests that may be conducted include ultrasound imaging and angiography. Ultrasound imaging is useful in finding lesions along the leg, however, it is heavily operator dependent, especially with smaller vessels below the knee and ankle [12]. Angiography can also be used to monitor patients after surgery. Digital subtraction angiography is an invasive diagnostic method that requires the use of a nephrotoxic contrast agent and ionizing radiation (X-rays) for imaging. All of these characteristics limit its use for frequent monitoring of disease progression [13, 14]. Magnetic resonance imaging (MRI) is expensive and requires the administration of Gadolinium, which is contraindicated in patients with renal insufficiency, who are often diabetic [15]. Hence it is highly desirable to have an easy-to-use, non-invasive, and inexpensive technique for diagnosis and monitoring of PAD before symptoms appear.

In clinical pilot studies, dynamic vascular optical spectroscopy (DVOS) has shown promising potential diagnosing PAD [16]. DVOS employs diffuse light, at multiple wavelengths, which propagates through biological tissues and is then collected by multiple photodetectors. The quantity and distribution of the collected light depends on the internal optical properties of the tissue. Because of the wavelength-dependent variations in absorption coefficients for oxygenated hemoglobin (HbO) and deoxygenated hemoglobin (HbD), the concentrations of HbO and HbD can be reconstructed from the collected light [17]. The DVOS system has many advantages over traditional diagnostic techniques, as it is easy to use, non-ionizing, non-invasive, and low cost. Apart from its potential to detect PAD, DVOS has been used to explore hemodynamic responses in humans for many purposes including detecting breast cancer [18, 19], measuring brain activity [20], evaluating joint diseases [21, 22], and monitoring chemotherapy treatment for breast cancer [23, 24].

This study uses the DVOS system to examine hemodynamic responses in the feet of diabetic patients for the purpose of diagnosing PAD. We hypothesize that assessing these responses during controlled manoeuvres, like thigh cuff occlusions, will yield vital physiological data. It is anticipated that this data will greatly improve the diagnosis of PAD in this patient population by providing important information about their vascular health.

## Results

**Fig. 1:**
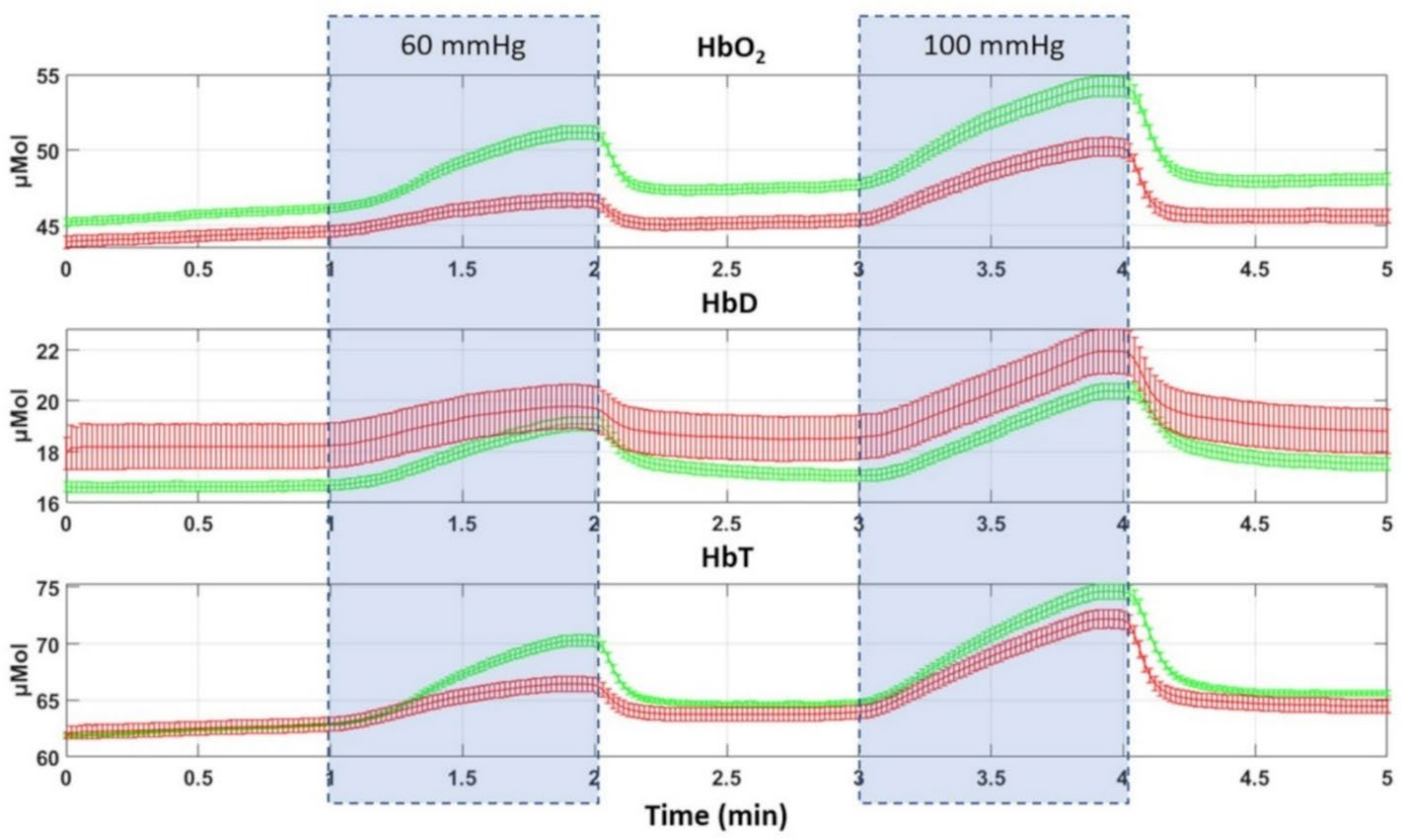
Group-averaged time traces (mean ± standard error) of HbO, HbD, and HbT during the thigh occlusion protocol for PAD (red) and non-PAD (green) participants. Shaded regions indicate the 60-mmHg and 100-mmHg cuff inflation intervals; unshaded regions correspond to baseline and recovery

Venous and partial arterial occlusions cause an increase in oxyhemoglobin (HbO), deoxyhemoglobin (HbD), and total hemoglobin (HbT) in less than 10 s after inflation of the thigh pressure cuff. Figure 1 shows the average time traces of HbO, HbD, and HbT, along with standard error bars, for PAD patients (red) and non-PAD subjects (green). During the 60-mmHg occlusion, concentrations of HbO and HbT increased gradually, whereas HbD showed a slight decrease in the non-PAD group and a modest increase in the PAD group. Following the cuff release at the 2-min mark, a significant decrease in HbO and HbT was recorded in both groups. A more pronounced pattern was observed during the 100-mmHg occlusion, characterized by greater separation between PAD and non-PAD curves, particularly in HbT and HbO profiles, highlighting the increased physiological impact of partial arterial compression.

The PAD group demonstrated decreased hemodynamic flexibility, characterized by reduced variations in HbO and HbT, alongside increased HbD levels during occlusion. The observed trends are physiologically reasonable, given that venous flow through the thigh is hindered during venous occlusion while arterial inflow continues. This results in a larger increase in HbO compared to HbD, reflecting the high proportion of HbO (> 90%) in arterial blood [25]. In addition, Fig. 1 highlights that the increases in HbO observed in healthy subjects were greater than the increases observed in the PAD patients, as detailed in Table 1.

**Table 1:** Group comparison and MLR results for the eight hemodynamic parameters

Parameter	Occlusion pressure (mmHg)	Mean (non-PAD) ± SD	Mean (PAD) ± SD	MWU p-vlaue	Adjusted OR (95% CI)	Parameter p-value	Sig. covariates (p < 0.05)
ΔHbO (µmol)	60	6.81 ± 4.11	2.01 ± 1.74	< 0.001	0.446 (0.360–0.553)	1.40E-13	Age
HF (µmol/s)	60	2.48 ± 0.85	0.86 ± 0.49	< 0.001	0.000387 (0.000021–0.00713)	1.24E-07	Age
VO_2_ (mL/100 mL/min)	60	0.066 ± 0.035	0.040 ± 0.022	< 0.001	0.480 (0.384–0.600)	1.31E-10	Age; smoking
Tp (s)	60	23.44 ± 9.04	20.70 ± 9.54	0.002	0.964 (0.933–0.996)	0.026	Age
ΔHbO (µmol)	100	9.44 ± 5.93	3.90 ± 3.55	< 0.001	0.696 (0.626–0.774)	2.26E-11	Age
HF (µmol/s)	100	2.90 ± 1.33	1.35 ± 0.90	< 0.001	0.129 (0.097–0.171)	3.85E-13	Age
VO_2_ (mL/100 mL/min)	100	0.079 ± 0.050	0.052 ± 0.029	< 0.001	0.661 (0.563–0.776)	4.26E-07	Age
Tp (s)	100	20.86 ± 7.09	17.12 ± 4.98	< 0.001	0.870 (0.817–0.926)	1.21E-05	Smoking

Adjusted OR (95% CI) and p-values for the hemodynamic parameter are from MLR adjusted for age, diabetes duration, hypertension duration, sex, BMI, and smoking status. “Significant covariates” lists adjustment variables with p < 0.05 in the corresponding model

Values are mean ± SD. MWU: Mann–Whitney U test (PAD vs non-PAD)

**Fig. 2:**
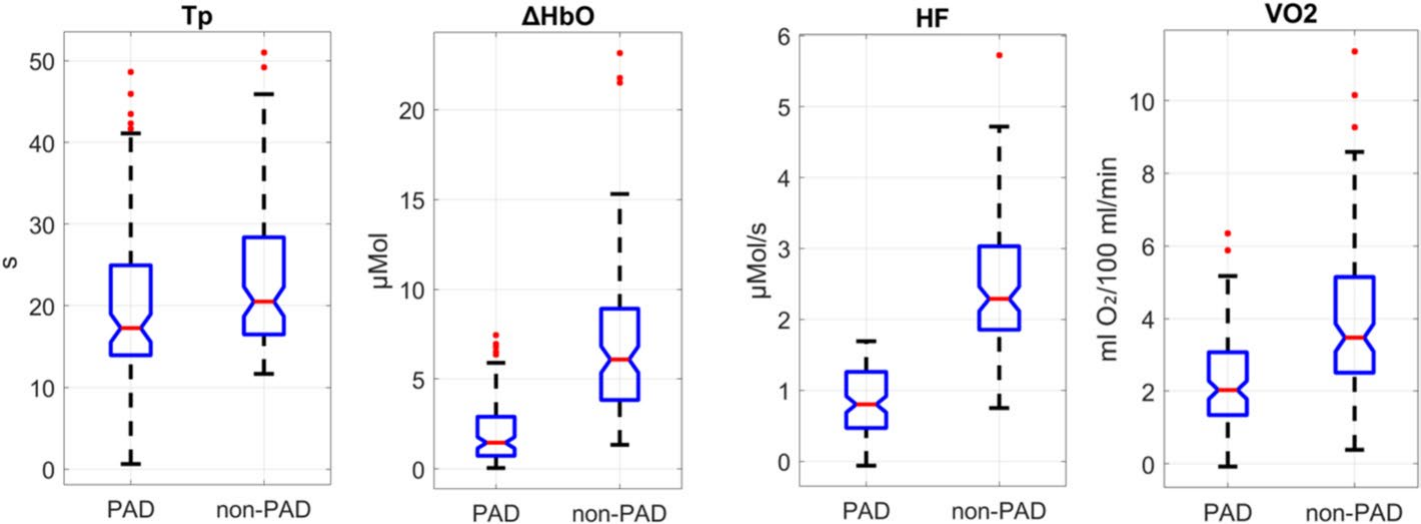
Box plots showing the distribution of TP, ΔHbO, HF, and VO_2_ for participants with PAD and without PAD. Red lines represent medians, blue boxes indicate interquartile ranges, the whiskers indicate the non-outlier range, and red dots denote outliers

Figure 2 shows boxplots for the four parameters derived from the hemodynamic time traces quantifying blood perfusion during a 60-mmHg occlusion. The figure compares hemodynamic parameters between PAD patients and non-PAD individuals across four different parameters: plateau time (TP), change in oxygenated hemoglobin concentration (ΔHbO), hemoglobin flow (HF), and oxygen consumption (VO_2_). There is a noticeable difference between the PAD and non-PAD groups in all parameters, indicating significant physiological differences related to the presence or absence of PAD. The ΔHbO and HF parameters show higher medians in non-PAD subjects, representing better vascular function compared to PAD patients (p < 0.01). No significant differences (p > 0.01) were found in TP, ΔHbO, HF, and VO_2_ between males and females (data not shown).

Table 1 details the mean, standard deviation, and statistical significance (p-value) of each parameter, as determined by the Mann–Whitney U test. Adjusted associations were evaluated using multivariable logistic regression (MLR) models (one model per parameter) controlling for age, diabetes duration, hypertension duration, sex, BMI, and smoking status, and are reported as odds ratios (OR) with 95% confidence intervals. Based on a significance threshold of p < 0.01, diabetes duration, hypertension duration, sex, BMI were not significant covariates in any model. Age was significant in all models except Tp at 100 mmHg, and smoking status was significant for VO_2_ at 60 mmHg and Tp at 100 mmHg.

Table 2 presents fivefold cross-validated performance for every parameter, including AUC, accuracy, sensitivity, specificity, and F1-score. All of them after controlling for age, DBT duration, and HTN duration, sex, BMI, and smoking status. Threshold-dependent metrics were computed using a fixed probability threshold of 0.50 (p ≥ 0.50 classified as PAD). ROC curve analysis reveals that HF from the 60-mmHg occlusion has the highest AUC 0.9833 ± 0.0089 and accuracy of 0.9181 ± 0.0351. This demonstrats its efficacy in differentiating between PAD and non-PAD subjects in this cohort. Additionally, ΔHbO from the 60-mmHg occlusion and HF from the 100-mmHg occlusion also show promise, with AUC of 0.9087 ± 0.0681 and 0.8457 ± 0.0729, respectively.

**Table 2:** Classification performance (fivefold cross-validation) of MLR models using each hemodynamic parameter (ΔHbO, HF, VO_2_, Tp) at 60 and 100 mmHg. Models were adjusted for age, diabetes duration, hypertension duration, sex, BMI, and smoking status. Values are reported as mean ± SD across folds

Adjusted Parameter	Occlusion Pressure (mmHg)	CV AUC (mean ± SD)	CV Accuracy (mean ± SD)	CV Sensitivity (mean ± SD)	CV Specificity (mean ± SD)	CV Precision (mean ± SD)	CV F1-score (mean ± SD)
ΔHbO	60	0.9087 ± 0.0681	0.8170 ± 0.0910	0.8551 ± 0.0837	0.7790 ± 0.1139	0.7980 ± 0.1025	0.8245 ± 0.0867
HF	60	0.9833 ± 0.0089	0.9181 ± 0.0351	0.9319 ± 0.0371	0.9043 ± 0.0364	0.9083 ± 0.0349	0.9199 ± 0.0343
VO_2_	60	0.8120 ± 0.0918	0.7447 ± 0.1204	0.7623 ± 0.1665	0.7290 ± 0.1030	0.7331 ± 0.1106	0.7446 ± 0.1280
Tp	60	0.6154 ± 0.0867	0.6058 ± 0.0425	0.6148 ± 0.0825	0.5983 ± 0.0610	0.5909 ± 0.0422	0.6007 ± 0.0526
ΔHbO	100	0.8457 ± 0.0729	0.7702 ± 0.0788	0.8225 ± 0.1378	0.7199 ± 0.1866	0.7671 ± 0.1167	0.7799 ± 0.0663
HF	100	0.8926 ± 0.0202	0.8000 ± 0.0389	0.8261 ± 0.0307	0.7739 ± 0.0567	0.7864 ± 0.0459	0.8054 ± 0.0350
VO_2_	100	0.7413 ± 0.0890	0.6851 ± 0.0696	0.7370 ± 0.1125	0.6355 ± 0.0699	0.6667 ± 0.0548	0.6979 ± 0.0728
Tp	100	0.7138 ± 0.0706	0.6702 ± 0.0524	0.7067 ± 0.0793	0.6381 ± 0.1319	0.6584 ± 0.0759	0.6762 ± 0.0433

*CV* cross-validation, *AUC* area under the ROC curve

Sensitivity = recall. Metrics were computed on held-out folds and summarized across 5 folds. Sensitivity, specificity, precision, and F1-score were computed using a fixed probability threshold of 0.50 (p ≥ 0.50 classified as PAD; p ≤ 0.50 classified as non-PAD).

It is also worth noting that the change in total hemoglobin (ΔHbT) was 7.89 ± 4.15 µmol/L for non-PAD and 3.62 ± 2.63 µmol/L for PAD at 60 mmHg, and 11.62 ± 5.84 µmol/L for non-PAD and 6.77 ± 3.98 µmol/L for PAD at 100 mmHg. However, due to the strong and statistically significant correlation between ΔHbT and ΔHbO (r > 0.7, p < 0.001), only ΔHbO is reported in Table 1 to avoid redundancy.

**Fig. 3:**
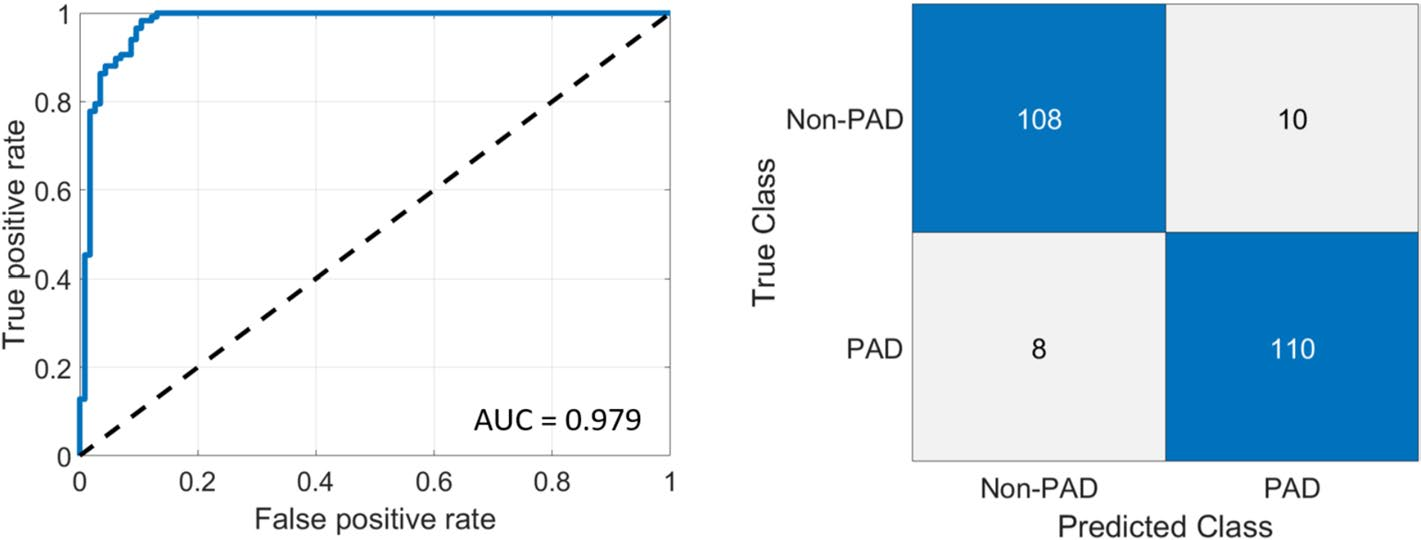
Out-of-fold ROC curve (left) and out-of-fold confusion matrix (right) for PAD classification using adjusted HF during the 60-mmHg occlusion. Out-of-fold predicted probabilities were obtained using stratified fivefold cross-validation from a MLR model adjusted for age, diabetes duration, hypertension duration, sex, BMI, and smoking status. The confusion matrix uses the same out-of-fold probabilities with a fixed threshold of 0.50 (probability < 0.50 non-PAD; probability ≥ 0.50 PAD)

Figure 3 presents the ROC curve and corresponding confusion matrix for PAD classification using the HF-based multivariable logistic regression model during a 60-mmHg occlusion. Predicted probabilities obtained from stratified fivefold cross-validation are shown as an out-of-fold ROC curve and out-of-fold confusion matrix. The ROC curve plots the true positive rate (sensitivity) against the false positive rate (1—specificity) at various threshold settings. The confusion matrix summarizes the number of correct and incorrect predictions made by the diagnostic test, compared with the true classifications. The model correctly classified 108/118 non-PAD and 110/118 PAD participants (10 false positives and 8 false negatives).

Adding medication indicators (statin, antiplatelet, and antihypertensive therapy) in a sensitivity analysis did not materially change conclusions; for HF at 60 mmHg, performance remained high (AUC = 0.9479; accuracy = 0.9440; sensitivity = 0.9402; specificity = 0.9478; F1 = 0.9442).

## Discussion

This study assessed the diagnostic utility of hemodynamic parameters derived from dynamic vascular optical spectroscopy (DVOS) in diabetic individuals with and without peripheral arterial disease (PAD). Three parameters (ΔHbO, HF, and VO_2_), particularly during 60-mmHg venous occlusion, demonstrated strong discriminatory ability. The findings presented in Table 2 indicate HF during the 60-mmHg occlusion achieved the strongest diagnostic performance in this cohort. The cross-validated accuracy, sensitivity, and specificity of HF during the 60-mmHg were high (> 0.9).

In the supine position, the venous pressure in the calf muscle hovers around 30 mmHg [25]. Applying a 60-mmHg cuff pressure to the thigh is generally sufficient to occlude venous outflow from the foot [26]. This creates a hemodynamic environment suitable for measuring changes in oxygenation and blood volume. Our study demonstrated that the 60 mmHg occlusion pressure produced higher cross-validated diagnostic performance compared to 100 mmHg. The stronger performance at 60 mmHg compared to 100 mmHg supports the concept that venous occlusion combined with preserved arterial inflow may provide a robust physiological challenge for assessing distal hemodynamics.

The higher ΔHbO and HF observed at 100 mmHg compared to 60 mmHg in Table 1 reflect greater blood pooling during the higher-pressure occlusion. This can occur despite partial preservation of arterial inflow. Systolic ankle pressures in diabetic patients commonly exceed 120 mmHg, allowing continued arterial entry during the higher-pressure occlusion (100 mmHg). This pattern is consistent with venous occlusion plethysmography findings showing greater blood pooling at 80–100 mmHg [27]. Near infra-red spectroscopy (NIRS) studies reporting larger hemoglobin changes at 100 mmHg due to persistent arterial inflow under partial occlusion [28].

The decision to focus on ΔHbO, HF, and VO_2_ was rooted in their established physiological relevance. VO_2_ reflects local metabolic demand and oxygen consumption, HF captures microvascular blood flow, and ΔHbO indicates oxygen availability in tissues. Together, they offer complementary insights into oxygen delivery and usage [29–31]. This multimodal approach enhances diagnostic robustness, especially when distinguishing between patients with and without PAD.

Toe pressures and TBI were not collected in this study. TBI is known to outperform ABI in diabetic patients who have non-compressible arteries. Future DVOS studies should include TBI to allow direct comparison and to validate DVOS performance against a more sensitive reference standard for distal PAD.

PAD is commonly accompanied by comorbidities and medication use that may influence physiological measurements. In this study, potential confounding was addressed through both cohort definition and multivariable modeling. The cohort was restricted to adults with type 2 diabetes and exclusions were applied for conditions/procedures likely to confound hemodynamics (e.g., COPD and recent lower-limb revascularization). In the primary analysis, each parameter (ΔHbO, HF, VO_2_, and Tp) was evaluated using multivariable logistic regression adjusted for age, diabetes duration, hypertension duration, sex, BMI, and smoking status. A sensitivity analysis additionally adjusting for statin, antiplatelet, and antihypertensive therapy yielded consistent results, supporting robustness to medication-related confounding. Residual confounding cannot be fully excluded. However, the consistency of results across adjusted and sensitivity models suggests the stability of the observed associations.

Our findings are consistent with prior work. Malagoni et al. investigated the use of NIR spectroscopy to measure VO_2_ in the calves of PAD and healthy individuals [32]. They reported a VO_2_ value of 0.052 ± 0.030 mL O_2_/100 mL/min for PAD patients, which closely matches the average VO_2_ observed in our study during 60-mmHg occlusion (0.040 ± 0.022 mL O_2_/100 mL/min). For non-PAD participants, Malagoni et al. reported a value of 0.047 ± 0.028 mL O_2_/100 mL/min, while our study yielded a slightly higher estimate of 0.0662 ± 0.0350 mL O_2_/100 mL/min. However, their study did not identify a significant difference between PAD and non-PAD groups. The discrepancy may be partly due to limitations such as a small control sample (30 healthy vs. 119 PAD participants), substantial age disparity (33.8 vs. 71 years), and differences in the measurement site (calf vs. foot).

Similarly, Wolf et al. examined hemodynamic responses in the calf using NIRS during 60-mmHg occlusion in three groups: 8 PAD patients, 8 subjects at risk of PAD, and 8 healthy subjects [33]. They found significant differences in VO_2_ and ΔHbO among the groups but no significant differences in HF. In contrast, our study, which involved a larger sample of diabetic participants (all non-PAD participants were diabetic), showed significant differences across ΔHbO, HF, and VO_2_, reflecting the effectiveness of our chosen parameters and measurement location.

Khalil et al. conducted a study using vascular optical tomographic imaging on 40 participants and found significantly larger HbT increases in non-PAD subjects during 60-mmHg occlusion [16]. Our findings of larger ΔHbO and HbT increases in non-PAD individuals compared to PAD patients, further reinforcing the use of these measures in PAD.

Some participants presented with leg ulcers, their inclusion reflects the real-world spectrum of PAD severity observed in diabetic clinics. It was not intended to imply that DVOS should be used as a screening tool in advanced disease.

Regarding clinical feasibility, the DVOS protocol used in this study takes about 10 min per session, including patch placement, cuff inflation, and data acquisition. None of the participants reported discomfort or pain during the 60 and 100 mmHg cuff occlusion. The system requires minimal operator training and can provides immediate diagnostic outputs on a screen.

This study has several limitations that should be acknowledged. First, duplex ultrasound was not performed systematically in all non-PAD participants. While high-risk individuals, those with symptoms, abnormal ABI values, or inconsistent findings underwent imaging, subclinical PAD cannot be completely excluded, especially in diabetic patients. Normal ABI does not always rule out PAD in diabetic patients. Second, the cohort was drawn from diabetic clinics in KAUH, and this single-center design may limit generalizability. Although the sample size (n = 236; 118 PAD and 118 non-PAD) is comparable to prior clinical feasibility studies, larger multi-center cohorts are needed to confirm robustness across broader patient populations. Third, DVOS is sensitive to movement. Less than 2% of DVOS recordings were excluded due to motion artifacts. This remains a practical limitation. Fourth, the study was cross-sectional and did not include longitudinal follow-up; therefore, the prognostic value of DVOS-derived parameters could not be evaluated. Finally, toe pressures and TBI were not collected. Incorporating ABI and TBI in future studies will allow evaluation of DVOS against the most appropriate diagnostic comparators for diabetic PAD.

Future studies could combine ECG-derived heart rate variability and entropy measures with DVOS-derived hemodynamic features [34]. This multimodal approach may capture complementary autonomic and peripheral vascular information, potentially improving PAD screening and risk stratification in diabetic patients.

## Conclusions

This study suggests that a low-pressure (60 mmHg) occlusion protocol using DVOS can differentiate between diabetic individuals with and without PAD in this cohort. Hemodynamic parameters (ΔHbO, HF, and VO_2_) showed strong diagnostic potential for PAD after adjustment for major clinical covariates. These findings are promising, but DVOS should currently be considered an adjunctive rather than a standalone diagnostic tool. Larger, multi-center prospective studies that incorporate TBI and systematic vascular imaging are needed to validate these results and establish clinical utility.

## Methods

This section describes the elements of the study design, including participant eligibility criteria, measurement instrument, clinical protocol, and data analysis procedures.

### Participant selection and eligibility criteria

A non-blinded prospective cohort study was conducted at King Abdullah University Hospital (KAUH) in Irbid, Jordan. The clinical study and optical measurements were conducted between August 2021 and June 2024. The study adhered to the principles of the Declaration of Helsinki and received ethical approval from the KAUH Institutional Review Board under protocol number 40/140/2021. KAUH serves a heterogeneous population exceeding one million individuals in northern Jordan and functions as a principal referral center for advanced medical care in the region.

Study participants were diabetic patients receiving healthcare at KAUH’s Diabetic Clinics, selected via convenience sampling from the patient lists of three physicians. Eligible participants were required to be 21 years of age or older, have type 2 diabetes, not be pregnant, and be free from chronic obstructive pulmonary disease (COPD). Although KAUH serves a broad regional population, this study specifically targeted diabetic patients attending specialized clinics to ensure that the selected sample size aligns with established practices for clinical studies evaluating advanced diagnostic technologies.

DVOS measurements were not performed on patients who had recently undergone lower-limb revascularization, as postoperative hemodynamics may be unstable and could confound interpretation of the optical signals. Participants exhibiting significant leg muscle contractions or excessive movement during data collection were excluded from the final analysis.

Type 2 diabetes was defined according to American Diabetes Association Standards 2024 criteria as having a fasting glucose ≥ 126 mg/dL, HbA1c ≥ 6.5%, or random glucose ≥ 200 mg/dL with symptoms of hyperglycemia [35].

A total of 241 diabetic patients were initially enrolled, all of whom underwent DVOS measurements. Of these, five subjects were excluded due to excessive motion artifacts, resulting in a final analytic cohort of 236 subjects. As shown in Table 3, 118 of these participants were confirmed to have PAD. Categorical variables are reported as counts and percentages, while continuous variables are expressed as mean ± standard deviation. This comparison highlights group differences relevant to disease status.

**Table 3:** Clinical and demographic characteristics of participants, categorized by PAD and non-PAD subjects. For continuous variables, mean ± standard deviation is shown. For categorical variables, the number of subjects and their percentage of the total number of subjects in the group are presented

Factor	Non-PAD (n = 118)	PAD (n = 118)	p-value (Mann–Whitney U test)
HTN (n,%)	65 (55%)	84 (71%)	–
DBT duration (yarr)	10.8 ± 9.5	13.5 ± 8.4	0.002
HTN duration (year)	5.4 ± 6.9	8.0 ± 8.5	0.009
Comorbidities^a^ (n, %)	43, 36%	48, 41%	–
Hyperlipidemia (n, %)	71, 60%	84, 71%	–
UIcers (n, %)	0, 0%	23, 19%	
Claudication duration (year)	–	4.3 ± 3.9	
Male (n, %)	52, 44%	50, 42%	–
Female (n, %)	66, 56%	68, 58%	–
Age (yr)	54.8 ± 13.7	59.1 ± 7.9	0.016
BMI (kg/m^2^)	30.3 ± 5.9	31.7 ± 5.5	0.071
Left ankle-brachial index	1.09 ± 0.08	0.88 ± 0.14	< 0.001
Right ankle-brachial index	1.10 ± 0.07	0.92 ± 0.11	< 0.001
Systolic blood pressure (mmHg)	136.51 ± 17.25	139.86 ± 11.89	0.478
Diastolic blood pressure (mmHg)	74.81 ± 9.88	72.43 ± 8.77	0.402
Tobacco use (n, %)	35, 30%	22, 19%	–
Anticoagulants (n, %)	12, 10%	14, 12%	
Antiaggregants (n, %)	80, 68%	105, 89%	
Statins (n, %)	70, 59%	75, 64%	
Antihypertensive (n, %)	65, 55%	84, 71%	

*DBT* diabetic, *HTN* hypertension, *BMI* body mass index

^a^Comorbidities include myocardial infarction, coronary artery disease, cerebrovascular diseases, lung disease, lower limb revascularization.

PAD patients exhibited longer durations of diabetes and hypertension, as well as higher average age, compared to non-PAD individuals. Specifically, hypertension duration was significantly longer in the PAD group (8.0 ± 8.5 years) than in the non-PAD group (5.4 ± 6.9 years, p = 0.009). Diabetes duration also differed significantly, averaging 13.5 ± 8.4 years in PAD patients versus 10.8 ± 9.5 years in non-PAD patients (p = 0.023). Age was significantly higher in the PAD group (59.1 ± 7.9 years) compared to the non-PAD group (54.8 ± 13.7 years, p = 0.003). These findings suggest that the presence of PAD may be associated with longer exposure to chronic comorbidities. The ankle–brachial index (left and right) was significantly higher in non-PAD patients compared to PAD patients. The blood pressures (systolic and diastolic) were not significantly different between PAD and non-PAD.

Following American Heart Association recommendations for PAD diagnosis, vascular experts evaluated each study participant to identify PAD in the diabetic patients included in the study. PAD symptoms assessed in this study included persistent intermittent claudication lasting more than six months, non-healing sores or ulcers on the legs or feet, calf muscle pain either at rest or during exertion, cold skin, reduced pulses, or obvious color changes. Normal ABI values (1–1.3) for individuals who did not exhibit PAD symptoms and who were not at high risk (age > 60 years, or age > 50 and > 10 years of diabetes and smoking history of ≥ 5 years), and no imaging evidence of lower-extremity arterial disease in the medical record were classified as non-PAD.

An ABI ≤ 0.9 indicated PAD and a value of ≥ 1.3 indicated arterial calcification. High-risk individuals, those with normal ABI but with one or more PAD symptoms, or those with arterial calcification (ABI > 1.3) were extensively examined with Duplex ultrasound/CT/MRA to verify the diagnosis. A stenosis that reduces the arterial diameter by ≥ 50% was considered as evidence of PAD. Participants with suspected PAD completed the Peripheral Artery Questionnaire (PAQ), a validated, disease-specific instrument that quantifies physical limitations, symptoms, social function, treatment satisfaction, and quality of life [36]. The PAQ scores were considered alongside clinical findings to confirm PAD diagnosis.

The 118 non-PAD volunteers, who were diabetic patients visiting the Diabetic Clinics for routine checkups and medication refills were classified as non-PAD based on the above classification criteria. All participants took part voluntarily and provided written informed consent, as approved by the KAUH Institutional Review Board (IRB).

### Instrument

**Fig. 4:**
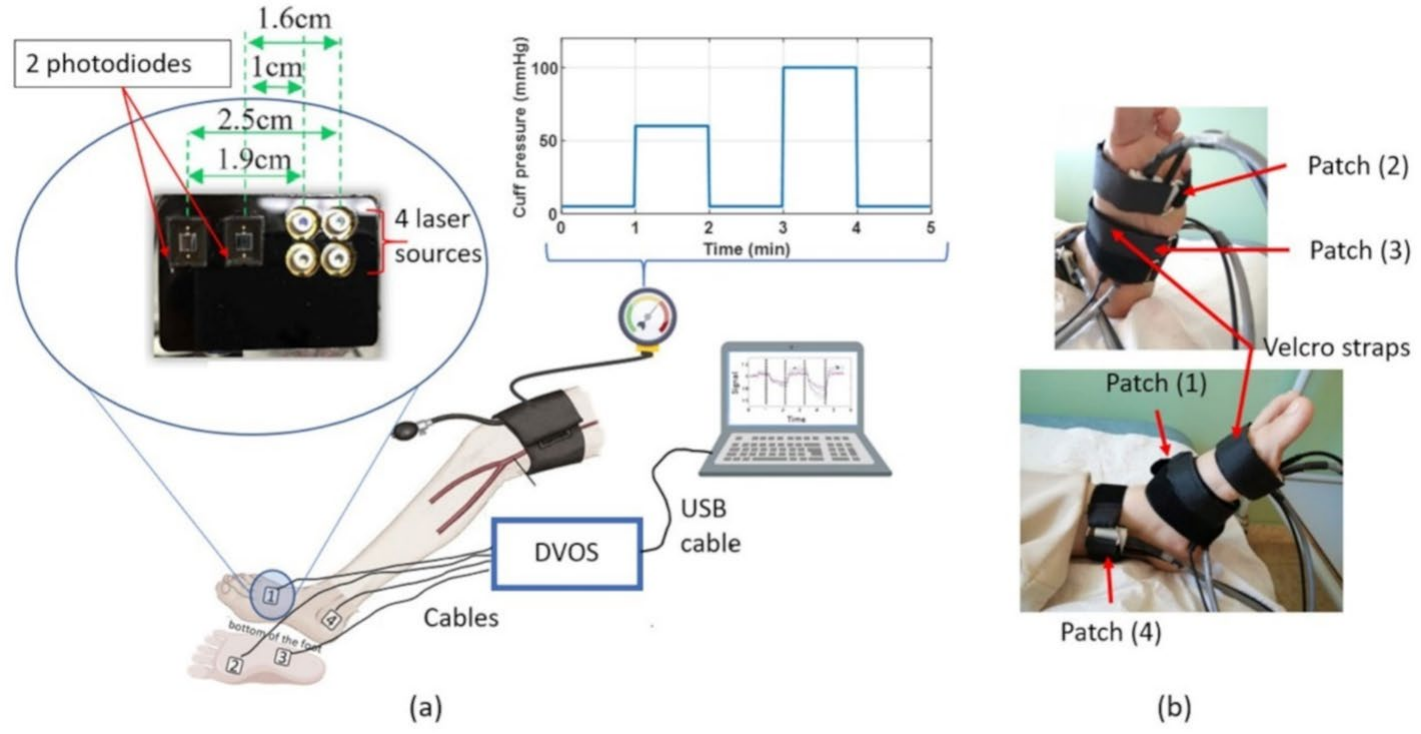
Overview of the dynamic vascular optical spectroscopy (DVOS) system and foot patch placement. a Schematic of the DVOS measurement setup, including a close-up of one optical patch (four laser diodes and two photodiodes), the thigh cuff, the DVOS acquisition unit, and the cuff-pressure protocol. b Representative photographs showing the placement of the four patches on the foot, secured with Velcro straps

The investigational clinical instrument used in this study was a low-cost, portable dynamic vascular optical spectroscopy (DVOS) device, previously described by [37]. Its main features are summarized here. The device consists of four patches placed on different locations of the foot. Each patch contains four light sources and two silicon photodetectors, as shown in Fig. 4a. The light sources are laser diodes emitting 5 mW at four wavelengths: λ = 670, 780, 808, and 850 nm. Figure 4 illustrates a schematic of the DVOS system, the measurement cuff-pressure, a close-up view of one patch, and an example of patch placement on the foot. The data acquisition rate was 2.56 scans per second, based on time-multiplexed measurements with automated gain adjustment. The four patch locations correspond to four distinct angiosomes, each supplied by a major artery (medial plantar, lateral plantar, dorsalis pedis, and posterior tibial, in this study). The emitted laser light interacts with the tissue beneath the patch and is detected by the photodetectors, enabling the system to monitor hemodynamics across these four angiosomes. The depth range of the hemodynamic measurements extended from 0.8 to 1.25 cm beneath the tissue surface.

### Measurement protocol

For the measurements, patients lay on their backs on a surgical bed in the Semi-Fowler position, with the head and trunk elevated to 30° and legs extended straight at the knee. After laying down, patients rested for about 10 min in a quiet room with a controlled temperature (21–23 °C). During this period, the blood pressure was monitored using an automatic blood pressure monitor (Omron HEM-7131-E, Kyoto, Japan). The ABI for each leg was measured and calculated as the ratio of the highest systolic blood pressure recorded at the ankle (from either the dorsalis pedis or posterior tibial artery) to the highest systolic blood pressure recorded in either brachial artery. The four DVOS patches were then placed directly on four major angiosomes of the foot. The patches were held in place using Velcro straps, as shown in Fig. 4b. A pressure cuff was wrapped around the thigh of the measured foot to produce a controlled vascular response, as presented in the upper right plot of Fig. 4a. The measurement protocol consisted of five one-minute periods: baseline with pressure less than 5 mmHg, 60-mmHg occlusion, recovery with pressure less than 5 mmHg, 100-mmHg occlusion, and second recovery. For safety and physiological reasons, the 60 mmHg occlusion was always performed before the 100 mmHg occlusion to ensure that the initial hemodynamic challenge remained limited to venous restriction without inducing arterial ischemia. A one-minute recovery period was implemented between the two tests to allow tissue perfusion to return to baseline, minimizing any carryover effects before the higher-pressure occlusion.

Although the cuff was placed on the thigh, the DVOS response reflects distal arterial and microvascular function because blood pooling during occlusion depends on downstream vascular integrity. This approach aligns with established methods, such as venous occlusion plethysmography [38, 39] and post-occlusive reactive hyperemia [40, 41], that use proximal occlusion to assess distal vascular responses.

The 60 mmHg and 100 mmHg inflation levels were chosen to assess hemodynamic changes, as these pressures allow for consistent venous occlusion while minimizing the risk of non-compressibility in patients with superficial femoral artery disease [42]. A cuff pressure of 5 mmHg or less was applied during baseline and recovery phases to maintain contact with the thigh without impeding venous blood flow. Cuff pressure was measured using a digital manometer (T68 Digital Manometer, Leaton, Shropshire, UK). DVOS data were collected continuously throughout all five periods. During measurements, subjects were instructed to remain still and refrain from talking or making gross movements. Three well-trained research assistants conducted the DVOS measurements under the specified conditions.

The 60-mmHg pressure cuff inflation induced predominantly a venous occlusion, and the 100-mmHg pressure cuff inflation induced both a venous occlusion and a partial arterial occlusion. The 60-mmHg pressure was above the venous pressure and below the diastolic pressure of all patients to guarantee that every vein was blocked without compromising the arteries. By inducing venous occlusion while leaving arteries open, we can isolate the blood flow originating from arteries. This approach enables the specific measurement of undisturbed arterial blood flow, which is crucial for assessing tissue perfusion and diagnosing PAD.

The higher pressure of 100 mmHg, which was above the diastolic and below the systolic pressure for most individuals (over 93%), induced a complete venous occlusion and partial arterial occlusion [43, 44].

### Signal processing and feature extraction

Signal processing entailed the removal of optical signals with low signal-to-noise ratio (SNR). SNR in the optical signals was calculated as the temporal mean over the baseline period (resting state), divided by the temporal standard deviation over the same period. Channels with SNR < 4 were excluded from the hemoglobin calculations. The selection of this threshold was determined through empirical analysis and visual inspection of the optical signal traces.

**Fig. 5:**
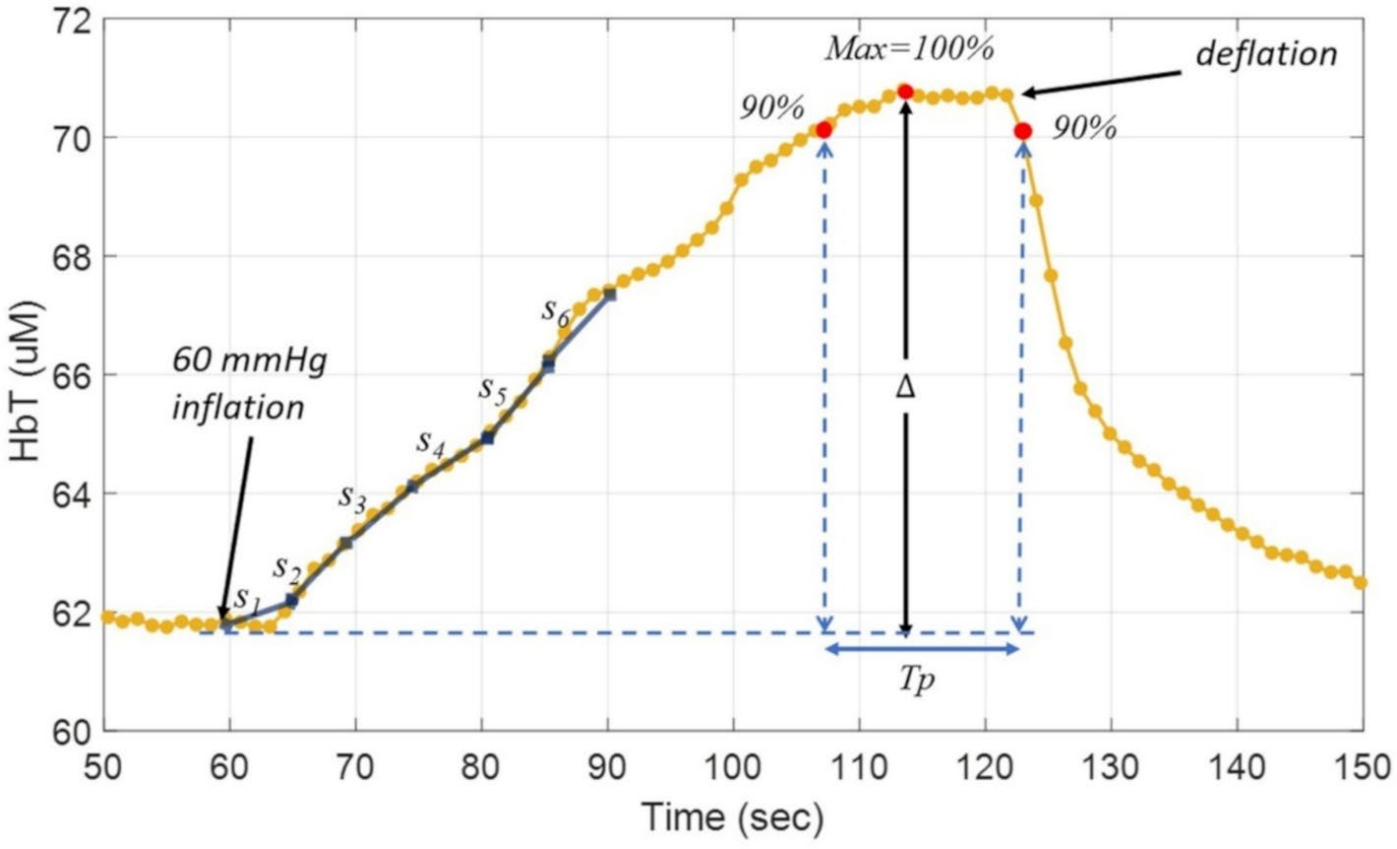
Typical time trace of total hemoglobin (HbT) during 60-mmHg occlusion. This figure illustrates the increase in HbT during a 60-mmHg occlusion. Hemoglobin flow (HF) is calculated as the maximum slope of the HbT rise over the initial segments (s − s₆), i.e., the steepest 5-s interval. The time trace shown represents data from the medial plantar angiosome patch of a 63-year-old male subject without PAD

Next, high-frequency noise in the included channels was attenuated using a 2nd-order low-pass digital Butterworth filter with a cutoff frequency of 0.51 Hz. The concentrations of oxyhemoglobin (HbO) and deoxyhemoglobin (HbD) were calculated using a partial differential equation-constrained multispectral reconstruction algorithm [45]. The total hemoglobin concentration [HbT] in tissue was calculated as the sum of HbO and HbD. As an example, Fig. 5 shows the HbT time trace from a patch located on the medial plantar angiosome of a 63-year-old non-PAD subject.

Four parameters were derived from the hemodynamic time traces (Fig. 5) to quantify blood perfusion [25]. As the signal-to-noise ratio in the medial plantar angiosome patch was the highest, this report specifically focuses on measuring the four parameters from the medial plantar angiosome patch. The four parameters are: plateau time for HbT (Tp), changes in HbO (ΔHbO), hemoglobin flow (HF), and tissue oxygen consumption (VO_2_).

ΔHbO was calculated by subtracting the mean value of HbO over 5 s before venous occlusion from the maximum value during venous occlusion, with units expressed in μmol.

HF was calculated from the rate of changes in HbT using Eq. (1):

1 $${\boldsymbol{H}}{\boldsymbol{F}}= {\boldsymbol{m}}{\boldsymbol{a}}{\boldsymbol{x}}\left(\frac{{\boldsymbol{d}}[{\boldsymbol{H}}{\boldsymbol{b}}{\boldsymbol{T}}]}{{\boldsymbol{d}}{\boldsymbol{t}}}\right).$$

The unit of HF is μmol/s. Since the arterial inflow depends venous compliance and may vary during venous occlusion, the first 30 s were subdivided into six intervals of 5 s each. The slope of HbT increase was calculated for each interval, as shown in Fig. 5. The highest rate of HbT increase (maximum slope) was used to estimate HF.

Additionally, VO_2_ was calculated from the rate of increase in HbD during the first 30 s after pressure cuff inflation. The increase in HbD during cuff inflation mainly reflects tissue oxygen consumption and the conversion of HbO into HbD [25]. VO_2_ was calculated using Eq. (2):

2 $${{\boldsymbol{V}}{\boldsymbol{O}}}_{2}={\boldsymbol{m}}{\boldsymbol{a}}{\boldsymbol{x}}\left(\frac{{\boldsymbol{d}}[{\boldsymbol{H}}{\boldsymbol{b}}{\boldsymbol{D}}]}{{\boldsymbol{d}}{\boldsymbol{t}}}\right).$$

The unit of VO2 is expressed as milliliters of O_2_ per 100 mL of tissue per minute (mL/100 mL/min), a convention widely adopted in the scientific community [25].

As shown in Fig. 5, plateau time (Tp) was calculated as the time, in seconds, for the HbT signal to pass from 90% of its maximum value before cuff deflation to 90% of the maximum value after the cuff starts to deflate. This study did not compute HRV or entropy metrics from RR intervals. Analyses focused on DVOS-derived hemodynamic parameters (ΔHbO, HF, VO_2_, and Tp).

All the signal processing and feature extraction were performed using MATLAB software (MATLAB 2023a; MathWorks Inc., Natick, MA, USA).

### Statistical analysis

In this study, we considered the four parameters presented above both for the 60-mmHg and the 100-mmHg cuff inflation, for a total of 8 parameters. The goal was to find the best parameters for distinguishing between the PAD group and the non-PAD group. To assess whether differences between these groups were statistically significant, the Mann–Whitney U test was applied for all continuous variables, as several did not meet the assumption of normality [46]. The Bonferroni correction was used to account for multiple comparisons and a threshold p-value of 0.00625 (= 0.05/8) was utilized to determine statistically significant differences between the PAD and non-PAD groups [47]. Additionally, we measured the correlation between pairs of parameters using Spearman’s rho coefficient to evaluate their interdependencies [46].

Multivariable logistic regression (MLR) was employed to examine the influence of each parameter on the likelihood of detecting PAD. MLR was used with PAD status (PAD vs non-PAD) as the dependent variable to adjust for relevant covariates: age, diabetes duration, hypertension duration, sex, BMI, and smoking status. Each hemodynamic parameter was evaluated in a separate model. Model discrimination and classification performance were evaluated using stratified fivefold cross-validation. In each fold, the model was trained on 4/5 of the data and evaluated on the held-out 1/5. Out-of-fold predicted probabilities from all folds were pooled to compute ROC curves and AUC. For threshold-dependent metrics (accuracy, sensitivity, specificity, precision, and F1-score), a fixed probability threshold of 0.50 was used (probability ≥ 0.50 classified as PAD; probability < 0.50 classified as non-PAD). Metrics are reported as mean ± SD across folds.

To assess potential confounding by medication use, a sensitivity analysis added binary indicators of statin, antiplatelet, and antihypertensive medication use to the multivariable models. Cross-validation, ROC/AUC analysis, and model implementation were performed in (MATLAB 2023a; MathWorks Inc., Natick, MA, USA), while other statistical analyses were performed using SPSS software, version 23.0 (IBM/SPSS Inc., Chicago, IL, USA).

## Data Availability

The datasets generated and analyzed during the current study are not publicly available due to privacy restrictions but are available from the corresponding author on reasonable request.

## Abbreviations

ABI: Ankle–Brachial Index
AUC: Area under the curve
DVOS: Dynamic vascular optical spectroscopy
HbO: Oxyhemoglobin
HbT: Total hemoglobin
HbD: Deoxyhemoglobin
HF: Hemoglobin flow
MLR: Multivariable linear regression
NIRS: Near infra-red spectroscopy
PAD: Peripheral arterial disease
ROC: Receiver operating characteristic
TBI: Toe–Brachial Index
TP: Plateau time
VO2: Tissue oxygen consumption
